# ACO (Asthma–COPD Overlap) Is Independent from COPD: The Case Against

**DOI:** 10.3390/diagnostics11071189

**Published:** 2021-06-30

**Authors:** Peter M. A. Calverley, Paul Phillip Walker

**Affiliations:** 1 Department of Clinical Science, University of Liverpool, Liverpool L9 7AL, UK; pmacal@liverpool.ac.uk; 2 Liverpool University Hospitals Foundation NHS, University of Liverpool, Liverpool L9 7AL, UK; 3 Department of Respiratory Medicine, Aintree Hospital, Lower Lane, Liverpool L9 7AL, UK

**Keywords:** COPD, asthma, asthma–COPD overlap, respiratory pathophysiology, bronchodilator reversibility

## Abstract

Over the last decade interest has been shown in people with symptomatic lung disease who have features both of COPD and asthma. In this review we examine how COPD and asthma are defined and examine clinical characteristics of people defined by researchers as having asthma-COPD overlap (ACO). We look at pathological and physiological features along with symptoms and consider the impact of each diagnosis upon therapeutic management. We highlight challenges in the diagnosis and management of airway disease and the various phenotypes that could be part of ACO, in so doing suggesting ways for the clinician to manage patients with features of both asthma and COPD.

## 1. Introduction

Chronic obstructive pulmonary disease (COPD) is now recognised to be a major cause of ill health, increased health care expenditure and premature mortality internationally [1]. The current definition of COPD advocated by the Global initiative for Obstructive Lung Disease (GOLD) highlights the importance of persistent airflow obstruction as a defining characteristic of this condition [2]. Clinically this presents a simple decision. Airflow obstruction is either present or it is not when the patient performs a technically satisfactory spirogram. However, the underlying biology of this apparently simple proposition is more complex.

Longitudinal studies measuring lung function prospectively and cross sectionally over time [3,4,5] have shown that both the FEV_1_ and FVC decrease with age and this is accelerated when people smoke tobacco or are exposed to other noxious inhaled insults [4,5,6]. Moreover, it is now clear that early life events impact significantly on lung growth and subsequent decline, resulting in a range of trajectories which the patient may follow up to the point where a diagnosis of COPD is confirmed by spirometry [7]. Traditionally, airflow obstruction is defined by the ratio of the FEV_1_ to FVC with a value of 0.7 or less signifying that obstructed airflow is present. This simple measurement identifies the presence of emphysema on CT scanning [8] and people at risk of accelerated lung function loss, at least in the earlier stages of COPD [9]. However, this ratio decreases with age and apparently healthy elderly people can be classified as having COPD based on this measurement [10]. This has led physiologists to propose that the lower limit of normal should be used to identify people where the ratio is below that expected by age [11]. This classifies people rather differently with more young people and fewer elderly ones being considered to have airflow obstruction. In practice, this changes relatively little at least in terms of the results of clinical trials [12] and there are now data suggesting that the fixed ratio of FEV_1_/FVC of 0.7 is the best predictor of subsequent ill health [13].

If it has proven difficult to define airflow obstruction, it has been even harder to decide what the term ‘persistent’ means. This term could imply that obstruction did not resolve when measured over time, but whether this could include significant improvements in lung function that were still below the normal predicted value, as is seen in some patients with chronic asthma, was not clear. These differences in interpretation were soon recognised as having therapeutic significance. In the 1990s an important paper from the Netherlands suggested that inhaled corticosteroids (ICS) could produce significant improvements in symptoms and lung function in COPD patients [14]. Subsequently these data were challenged, especially by physicians in the UK, who argued that the improvements seen were due to the inclusion of patients who would normally be diagnosed as having bronchial asthma. This led to an intense debate about how to best define bronchodilator reversibility in order to separate COPD from asthma. In Europe, a very tight definition of irreversible disease was proposed which precluded almost any lung function change after exposure to an inhaled bronchodilator [15]. This created a ‘Catch 22’ situation where any patient where lung function improved with treatment could not have COPD because treatment had improved their lung function! Such a tight definition is not used today but illustrates evolution over time.

As a result, rather than consider in more detail what bronchodilator reversibility might signify in a patient with structural lung damage due to cigarette (or any other relevant) exposure, the tendency has been to assign patients to mutually exclusive silos—either COPD or asthma. Clinicians have always realised that this is an oversimplification and that some typical COPD patients would show larger than expected benefit from treatment of various types. What has been less clear is whether this behaviour represents a variation within an established diagnosis or is a discrete condition which consistently behaves differently from ‘true’ asthma or COPD.

Over the last decade there has been renewed interest in the idea of an asthma–COPD overlap (ACO) state in part driven by the desire of the pharmaceutical industry to identify a subset of COPD patients who might respond better to the existing anti-inflammatory treatments and to explain why some asthmatic patients did not improve to the degree anticipated when given them. The most cogent rational academic exploration of this idea came from Gibson et al. in 2009 [16]. Subsequently there have been many publications reporting data in patients believed to be exhibiting ACO and suggestions have been made about how best to operationalise this concept [17,18,19]. In this review we will consider what has been proposed and outline our reasons for believing that ACO is not a helpful way to understand the variation seen in the way that disease develops in patients with asthma or COPD.

## 2. Defining ACO

A key issue limiting the usefulness of the ACO concept is the lack of a consistent definition. This not only hinders academic study but also confuses the clinician. This problem is not restricted to ACO but has bedeviled the field of ‘airways disease’ for the last 60 years. Indeed, the portmanteau term ‘airways disease’ to describe asthma, COPD and related conditions is itself unsatisfactory as it fails to account for airflow obstruction due to emphysema. Clearly if we have issues defining asthma and COPD, it is going to be hard to identify overlaps between them.

As has been noted before, defining both asthma and COPD is like love—everyone knows what it is when it happens, but it is hard to explain to other people. By the 1980s advances in pulmonary pathology and physiology meant that definitions based only on symptoms such as chronic bronchitis were superseded by approaches using structural and/or lung function criteria. The CIBA symposium in 1959, perhaps the most famous of the meetings which attempted to re-define these conditions, proposed definitions based on variability in lung function for asthma, the presence of enlarged airspaces due to tissue loss for emphysema and symptoms of chronic cough [20]. Helpful as these definitions were in providing a focus for further study, they contained a fundamental weakness, namely that each relied on a different domain—physiology, pathology or symptomatology—to characterise the disease, building in the study of overlap states from the outset.

In the 1970s and 1980s, attention was paid to whether chronic bronchitis or physiology, in the form of the FEV_1_, identified discrete natural histories of disease and whether this differed from that seen with patients diagnosed in life with emphysema. The famous longitudinal study of British postal workers led by Charles Fletcher provided the unexpected answer that it was lung function that identified individuals whose lung disease progressed with smoking, rather than the symptoms of bronchitis [21]. Thereafter symptoms were seen to be secondary to lung pathology identified by abnormal lung function rather than identifying a discrete condition. While this is likely to be true, the importance of symptoms like mucous hypersecretion as a marker for respiratory infection and exacerbation [22] and lung disease in the earliest phases of COPD [23] has been neglected until relatively recently.

The overlap between emphysema and bronchitis (clinically defined) seemed to have an international dimension with workers in the USA reporting most of their patients with chronic airflow obstruction as having emphysema (based on CXR appearances) while in Britain similar patients were defined as being bronchitic [24]. Eventually these semantic problems were resolved, but there was still a belief that patients with emphysema without bronchitis maintained normal arterial blood gas tensions while those reporting bronchitis were more likely be hypoxaemic [25]. Again, subsequent pathology studies showed that emphysema could be associated with hypoxaemia [26]. With hindsight it is likely that some of the ‘blue and bloated’ patients had undetected bronchiectasis and/or left ventricular dysfunction, but this illustrates the way in which ideas about airflow obstructive disorders has been refracted through the tools available for their study rather than any intellectual limitation of those leading the investigations.

The contrast between asthma and bronchitis was not immune from the debate between ‘lumpers and splitters’. Unlike the British who felt that chronic bronchitis was a discrete disorder of prognostic significance, the Dutch group in Groningen led by Dick Orie advocated the concept of chronic non-specific lung disease which recognised the heterogeneous nature of conditions others would describe as bronchitis, emphysema or asthma, and grouped them together [27]. In this approach we have the origin of the concept we now consider as ACO and, as noted already, it received considerable push back when the results of their clinical trial of inhaled corticosteroids was first published [14]. However, the conceptual framework developed in the Netherlands was taken up by Gordon Snider in Boston and led to his visual representation of COPD in a non-proportional Venn diagram which was adopted by the American Thoracic Society in its original Standards of Care for COPD document [28]. Thus, a potential for ACO was recognized, but its nature was not clarified.

Longitudinal studies in the Netherlands and New Zealand in young people who have the clinical and physiological characteristics of asthma have shown how over time they can develop fixed airflow obstruction which is often diagnosed as being COPD [29,30]. Whether these people have the same pattern of structural damage seen in typical smoking induced COPD is unclear as is their response to therapy. By contrast, much less information is available about whether people with typical COPD go on to develop disease features more typical of chronic asthma.

Although interest in this topic subsequently declined, the 2009 article by Gibson et al. reignited old uncertainties about whether a discrete phenotype of patients with features of both asthma and COPD existed [16]. These authors approached this from an asthmatic perspective and placed significant emphasis on the role of the bronchodilator response in identifying these patients, as well as emphasising the increased sputum neutrophilia seen in their ACO subjects compared with asthmatics and healthy older adults. Coming at a time of concerns about the risk of pneumonia developing in COPD patients treated with ICS, this approach offered a way of identifying a subgroup for which the benefit of ICS treatment was easier to justify.

In response to these concerns, the Global Initiative in Asthma (GINA) and Global Initiative in Obstructive Lung Disease (GOLD) produced a joint consensus document highlighting practical approaches to the management of ACO [31]. Subsequently the report of workshops convened by the ATS and ERS were published [19,32]. The GOLD/GINA approach was not to offer a specific set of criteria on which a diagnosis of ACO was based but to suggest that ACO could be considered when features usually considered typical of asthma or COPD were present in the same patient [31]. This group offered a series of choices to the clinician about clinical and laboratory features they felt were important, and more detail can be found on the respective websites. There was no attempt to weight the features for their relative importance, a task sensibly left to the individual clinician to decide, from what is basically advice on what to consider in managing patients presenting with atypical clinical findings. However, this level of individual decision makes it hard to draw conclusions about the nature and management of this condition and assumes that treatment approaches valid for the individual diseases are as effective in someone exhibiting these ‘overlap’ findings.

By contrast, the ATS workshop considered a wider range of issues and raised a series of research questions which needed to be addressed before the nature of ACO could be considered finalised [32]. The European consensus group reviewed the entry criteria used in a range of clinical trials of asthma and COPD and developed a series of major and minor diagnostic criteria summarised in Table 1. This group provided the clearest operational definition of ACO but, to date, this has not been widely accepted, with other groups adapting it to local perceptions of what the key features of ACO might be. The resulting plethora of reported definitions is summarised in the helpful review of Cazzola and Rogliani [33]. It is no surprise in this setting that the type of patients included in what are mainly observational studies appear to be rather different in their nature, illustrated by Barczyk et al. [34].

These problems in definition raise several concerns about the utility of the term ACO as an aid to both academic and clinical understanding of people with objectively defined airflow obstruction. In the following sections we will examine what evidence we have for a discrete overlap of pulmonary pathology between asthma and COPD, whether patients meeting the definition of ACO behave differently from others not diagnosed in this way and whether objective physiological tests which are often the main driver of an ACO diagnosis can be relied on to distinguish these patients from others with chronic airflow obstruction.

## 3. A Pathology of ACO?

There is a dearth of evidence for a discrete pathology occurring in ACO patients. This reflects the lack of a clear definition discussed above and the fragmented nature of the data about structural and immunological features of those who do meet whatever definition is considered appropriate. The issue is not just whether the pathologies typical of asthma or COPD co-exist in the same person, but in how many people such features are present without them exhibiting the defining conditions of the overlap state.

In most cases it is accepted that a prior clinical diagnosis of asthma indicates the continuing presence of that condition. However, this is not necessarily the case. Often the diagnosis is not confirmed by any objective measurement and, in the case of the overlap between asthma and obesity, an asthma diagnosis is often made in patients without any evidence of enhanced airway responsiveness or spontaneous fluctuation in lung function [35]. The clearest evidence for a common set of pathological characteristics in asthmatics has come from biopsy studies largely conducted in milder disease and autopsy data in the relatively few people who die from the disease. In most cases there are features of Th-2 inflammatory changes, increased numbers of eosinophils in the tissue and airway lumen and, as the disease worsens more neutrophils accumulate. A striking finding is the increase in bulk of the airway smooth muscle which helps explain several of the physiological features of the disease [36,37,38].

For many years there was a consensus based on chest X-ray studies that emphysema only rarely occurs in asthmatic patients but was a frequent finding in those presenting with COPD. It is now clear that in most COPD patients the loss of the small airways precedes the development of emphysema which becomes a more prominent feature as lung function loss worsens [39,40]. The advent of quantitative CT scanning has allowed the relationship between structure and function to be explored in life. One of the best studies is that of Hartley et al. who studied 171 asthmatics, 81 COPD patients and 49 healthy subjects [41]. Patients met standardised diagnostic criteria and were not classified as being ACO or non-ACO in nature. These workers found that airway wall thickness increased as FEV_1_ decreased in asthmatics, but the degree of air trapping, a measure of pulmonary hyperinflation, was the main driver of a low FEV_1_ in COPD patients. The degree of emphysema contributed to the decreased FEV_1_ in COPD patients but was infrequent in patients with asthma. Thus, different pathological changes contribute to the impaired physiology, but airways disease plays a role either directly or indirectly in both asthma and COPD.

These pathological issues have been more directly addressed by a Japanese group who report 3D CT imaging in COPD patients with and without a diagnosis of ACO based on the presence of a bronchodilator response and matched for their smoking history [42]. In this study an FEV_1_ change of more than 12% baseline and 200mL after an unspecified bronchodilator or 4 weeks of anti-inflammatory treatment together with variable symptoms were used to define ACO. Patients exhibiting a positive response had thicker proximal airways and less evidence of emphysema than those who did not. However, the mean FEV_1_ in this study was relatively high at 70% predicted, so extrapolation to more severe COPD should be done with caution.

Direct study of the nature of airway inflammation in ACO subjects should help resolve matters. One of the few studies to report data on this topic came from a group in Basel who systematically collected biopsies from 129 COPD patients without features of asthma, 19 smoking asthmatics and 18 COPD patients with ACO, all of whom were undergoing diagnostic bronchoscopy and biopsy procedures. They defined ACO using a modified ERS consensus definition [43], but unlike other studies the ACO group did not show greater reversibility to salbutamol that the non-ACO COPD patients. The ACO patients had higher exhaled breath nitric oxide concentrations, more blood eosinophils and significantly better lung function than the COPD control group. These differences in disease severity make it difficult to interpret the greater degree of basement membrane thickening seen in the ACO patients compared with the smoking asthmatics. As the authors comment, their data is preliminary and other focused studies will be needed to address the question of what kind of pathological changes occur in what patients.

An alternative approach to establishing overlap would be to look for differences in biomarkers of tissue inflammation between ACO and non-ACO COPD patients. This would be a very helpful strategy if the biomarkers concerned were both specific and sensitive in distinguishing asthma from COPD. Many inflammatory biomarkers have been linked to asthma with fractional exhaled breath nitric oxide (FeNO), being widely used as a marker of Th-2 inflammation. Unfortunately, the inflammatory process and its attendant biomarkers change as the clinical presentation of asthma evolves, with a more neutrophilic, less eosinophilic profile being seen in severe asthma, especially among patients who are relatively resistant to systemic corticosteroid treatment [44]. Blood eosinophilia is seen as a marker of airway eosinophilia, although studies where these variables have been directly compared suggest that this relationship is relatively weak [45] and there is little agreement about what constitutes eosinophilia and how best to express the data. Unsurprisingly, a raised peripheral blood eosinophil count is not required in the diagnosis of asthma [46]. Nonetheless, the peripheral blood eosinophil count does predict the response to biological treatments in severe asthma [47] and in general population samples of COPD sufferers, those with an eosinophil count as high as 350–600 cells /µL have an increased risk of hospitalisation [48].

Attempts to use these variables to separate ACO from COPD patients who do not meet the clinical criteria for this condition have generated conflicting results. Li et al. found that in 48 patients (42% with a history of smoking and 50% taking ICS) that an FeNO >31.5 ppb identified patients with ACO who smoked with a sensitivity of 70% and a specificity of 90% [49]. However, both the reproducibility of these threshold values and their predictive power need to be replicated in other cohorts. Nonetheless, there is a growing sense that patients who have a history of asthma before the age of 40 behave differently to those whose smoking related COPD develops later in life. Data from Spain suggests that the airway responsiveness is greater, peripheral blood eosinophil count is higher and serum IgE levels are higher in COPD patients with a prior diagnosis of asthma [50]. Further work on well characterised cohorts preferably with appropriate CT imaging should help clarify these relationships. However, the largest comparative cohort study to date, NOVELTY, found no difference in blood eosinophil counts between the asthma, COPD and asthma-COPD overlap groups that they recruited [51], suggesting that blood eosinophils are not useful discriminants in routine clinical practice in identifying what physicians felt constituted ACO.

In many ways the most powerful argument for the existence of an overlap state between asthma and COPD comes from genetics. By combining data from several pathological studies in asthma and COPD, Christenson et al. found that genes associated with a Th2 phenotype in asthmatics were also expressed in patients with COPD and that blood eosinophil counts and airway responsiveness were increased when this was the case [51]. They argue that these genes might be involved in the earlier stages of the development of COPD. However, it is important to recognise that the pathological changes associated with COPD differed from those seen with asthma, with the exception of the eosinophil numbers. Clearly these findings also merit replication in patients meeting any of the current ACO definitions.

## 4. The Clinical Significance of ACO

It could be argued that it is not important whether or not there is a clear definition of ACO if clinicians can identify a group of patients who should be managed differently. This approach runs the risk of committing the Procrustean crime of making the facts fit the prejudice of the observer—in this case that ACO must exist.

In Table 2 [52,53,54,55,56,57,58,59,60,61] we summarise some of the many studies which have looked at the clinical characteristics of ACO (defined in a variety of ways) in clinical populations which vary by country and care setting. The reported prevalence of the condition varies as does the sample size studied, ranging from 1.5% to 27.4% of populations with asthma or COPD. As noted by Spanish workers, the very strict definition of substantial bronchodilator reversibility change excludes so many patients that the definition had to be relaxed to allow them to identify anyone with ACO [54]. This approach feels like a very uncertain way of defining a disease as the higher threshold had originally been suggested as a way of avoiding random variation in a positive BDR (see below). There is an impression that patients identified as having ACO are somewhat younger, are more symptomatic and more likely to report exacerbations than COPD patients not identified in this way. This is supported by several of the review articles which have summarised the findings in these and/or other data sets [16,17,18,33,62]. Two further studies are worthy of note. In a validation of the ERS symptom score, Nelsen et al. found that most of the symptoms in the battery worked as well for COPD as for ACO, i.e., clinically the patients were very similar. However, wheeze seemed to differ and was not a reproducible symptom, suggesting that reliance on this complaint, at least in COPD patients, could be misleading [63]. A different approach was used by Pascoe who reported a mathematical analysis of a health symptom questionnaire in a large population of patients with obstructive lung disease. The resulting model was accurate in distinguishing asthma and COPD but the authors suggest that patients not falling into these groups are very heterogeneous and hard to classify [64]. This heterogeneity is emphasised by the results of the NOVELTY study [52]. Here over 11,000 patients entered an observational study based on their doctor diagnosed asthma, COPD or ACO. There was substantial heterogeneity across the diagnostic groups and physician determined disease severity classes showing that, in the ‘real world’ diagnostic groupings are not rigidly applied.

So far, data have largely focused on the overlap of COPD and asthma, i.e., in patients who look like they have COPD, how many have some features that are atypical and would fit better with a diagnosis of asthma. There are plentiful data about what happens when a young person diagnosed with asthma continues with symptoms into adulthood. Work from the Netherlands, Aberdeen, Australia and New Zealand have shown in patients followed for up to 45 years that a significant number of asthmatics go on to develop fixed airflow obstruction which is re-defined as COPD by the clinicians managing them [29,30,65,66,67]. In a recent report of children followed to age 45, a diagnosis of ACO based on the presence of airflow obstruction and a history of previous asthma irrespective of smoking history was made in an estimated 3% of the population and, like COPD without an asthma diagnosis, was especially likely to do so in those with the worst lung function at the age of 7 years [65]. These data provide further support for the early origins of COPD in a significant number of patients but ACO described here represents a different entity from the COPD with asthmatic features that has fueled much of the ACO debate [68]. It is now clear that tobacco smoking decreases the effectiveness of inhaled corticosteroid treatment in both asthma [69] and COPD [70], further complicating the distinction between COPD with asthmatic features and asthma with features of COPD in longitudinal studies like that of Bui et al. [65].

## 5. The Physiology of ACO

Thus far, physiological measurements made in ACO patients have been largely confined to spirometry rather than collecting data about lung volumes or gas transfer. Some studies have reported the results of non-specific bronchial challenge testing with either inhaled histamine or methacholine as the inhaled agonist [71,72], but most studies restrict themselves to reporting the results of a single bronchodilator reversibility test (BDR) usually using inhaled salbutamol as the test drug. The interpretation of this apparently simple test has proven to be fraught with difficulty, especially in patients with COPD and has been reviewed in detail on several occasions [73]. As these tests are often crucial in the clinician’s decision about whether the patient has ACO or COPD, it is important to consider them in some detail and to highlight why simple assumptions about how to interpret them can be misleading.

In routine laboratory practice both the measurement of airway hyperresponsiveness (AHR) and BDR rely on changes in the FEV_1_, the volume that a subject can expire in one second during a forced expiration from total lung capacity. Reliable standards exist for the performance [74] which exploits the development of flow-limitation during the manoeuvre to reduce between test variation. Nonetheless there is a short term and between day physiological variation in the FEV_1_, which means that tests repeated a few minutes apart can differ by chance by up to 160 mL. Rather surprisingly this between test variability is not much influenced by the initial FEV_1_ of the subject, although it is somewhat lower when the pre-test FEV_1_ falls below 1.5 L. By contrast the FVC is more effort dependent with a potential for more between test variation which has meant that it is less often reported during AHR and BDR tests. This is unfortunate as change in FVC gives more clinically relevant data about lung volume change in COPD and has been suggested as a better guide to AHR in asthma [75].

Although considered as being equivalent measurements of airway smooth muscle responsiveness, AHR and BDR tests are not interchangeable and often say more about the pathology of the surrounding lung than the medium sized airways where most of the inhaled stimulant is delivered. In general, AHR testing is used to diagnose asthma with a series of threshold changes identifying mild to severe degrees of airway irritability. This approach works well if the initial FEV_1_ is relatively normal, but as the pre-test FEV_1_ falls the same dose of agonist can produce a more dramatic fall in FEV_1_ due to the altered airway geometry rather than a greater degree of airway smooth muscle contraction. In this context, absence of AHR is more informative than its presence, as has been seen when trying to interpret the diagnosis of asthma in obese subjects [34]. Relatively few groups have looked at AHR in more severe COPD. When we did, we found that this was a surprisingly frequent occurrence [76] and accompanied by increases in end-expiratory lung volume, likely reflecting worsening flow limitation with the agonist drug. Although relevant to why such patients were more symptomatic and are prone to more exacerbations of COPD, we were confident that the changes we saw were related to predictable physiological changes in patients with more severe lung damage due to typical smoking-related COPD, as these patients had no pointer to a diagnosis of asthma, either currently or in their past. Structural differences may help explain the observation in mild to moderate COPD that those with the greatest AHR show the fastest decline in FEV_1_ over time [77].

The situation around interpreting bronchodilator responsiveness is, if anything, even more complex. Table 3 summarises some of the main issues that have emerged over several decades of applying this test in clinical practice. Unlike AHR testing, which examines the ease with which airway smooth muscle contraction can be induced, BDR testing looks at the effect of an inhaled drug that promotes airway smooth muscle relaxation (usually 4 puffs of salbutamol) to improve lung function over a short time, commonly 15 min. This is a satisfying test to conduct in a labile asthmatic patient where the FEV_1_ can increase by 500 mL or more and often returns to values within the predicted normal range. This form of acute reversibility is diagnostic of bronchial asthma when it occurs but is not the kind of change commonly seen in patients diagnosed as having ACO.

As with AHR testing, the physiological basis of BDR is more complex than is commonly appreciated. Airway smooth muscle (ASM) is widely present throughout the bronchial tree down to the terminal bronchioles. In health there is a normal spontaneous variation in the degree of airway smooth muscle activation (ASM tone) which can be reduced or abolished by bronchodilator drugs; hence the enthusiasm of endurance athletes to acquire a diagnosis of asthma. This spontaneous fluctuation in ASM tone is exaggerated in bronchial asthma through a combination of airway inflammation and enhanced ASM bulk [78] but is preserved in COPD. However, in these patients the baseline airway calibre is reduced and structural changes can increase the degree to which normal physiological changes in ASM translate into changes in airflow resistance which is being indirectly assessed by the FEV_1_. These effects are not as dramatic as is the case in bronchial asthma but are more than enough to account for the variable bronchodilator responses that characterise many COPD patients. None of this requires there to be any ‘co-existing’ asthmatic pathology in the lungs of the COPD patient.

**Figure 1:**
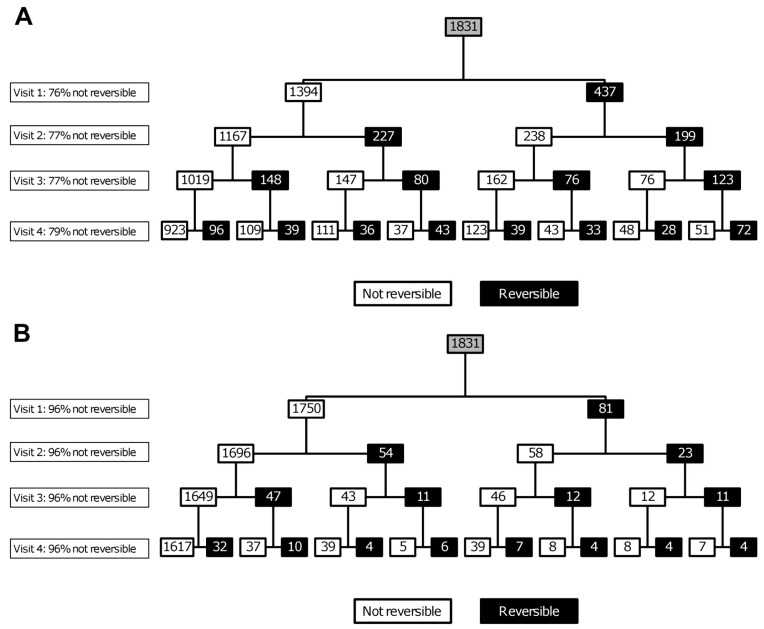
The reproducibility of the classification of bronchodilator reversibility in 1831 people with COPD who participated in the ECLIPSE cohort study. In (**A**) reversibility is defined by ≥12% and ≥200 mL increase from pre-bronchodilator FEV_1_ and (**B**) an absolute response of >400 mL from pre-bronchodilator FEV1 [82].

These theoretical considerations aside, there are many obstacles to the easy interpretation of a bronchodilator reversibility test. The protocol adopted will influence the result. In patients with moderate–very severe airflow obstruction the number of positive tests rises with the number of bronchodilators given to the patient [79], a fact clinically exploited in the use of long-acting inhaled dual bronchodilators [80]. There has been an extensive discussion about how to define a positive result. The simple approach of looking for a large percentage change from baseline works well if the pre-test FEV_1_ is relatively preserved, but a 160 mL increase in FEV1 which is within the spontaneous variability of two FEV_1_ measurements could be interpreted as 16% reversibility in a patient with a baseline FEV_1_ of 1 L. This led to the current recommended volume change which must be at least 12% of the baseline value and exceed 200 mL [81]. This was derived from basic principles and experience in population studies rather than empirical data from studies of COPD patients which helps explain its problems in clinical practice. Using a very large absolute difference of 400 mL between measurements to define a positive test greatly decreases the number of positive responses, but did not abolish the between visit fluctuation in classification in those who tested positive initially as shown in Figure 1 from the ECLIPSE study [82].

**Figure 2:**
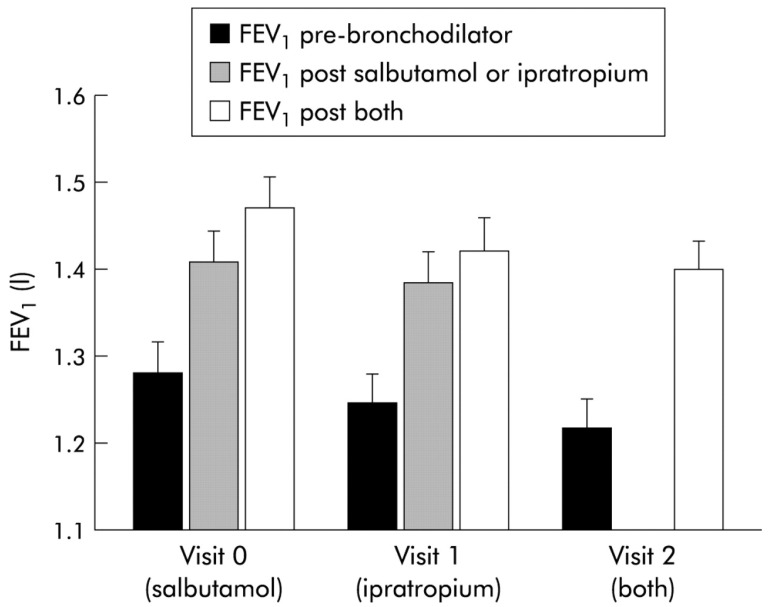
Response to bronchodilators in 660 people with COPD who participated in the ISOLDE study. The results presented show absolute FEV_1_ pre-bronchodilator and after administration of one or more bronchodilator. At visit 0 subjects received salbutamol followed by ipratropium bromide, at visit 1 ipratropium bromide followed by salbutamol and visit 2 where both bronchodilators were administered together [79].

To use any definition of bronchodilator reversibility to make clinical decisions requires it to be stable from day to day and this is not the case in patients without a history of asthma and diagnosed as having smoking-induced COPD. This became apparent when the reversibility testing data from the ISOLDE study conducted over 20 years ago were analysed [79] and has been confirmed in other large prospective clinical trial populations where carefully standardised reversibility testing was undertaken [82]. Figure 2 illustrates the problem. Over the 3 years of testing, significant numbers of individuals meeting the reversibility criteria at their first visit would be reclassified when tested on a subsequent visit. Overall, the percentage of people in the population testing positive at a given attendance was remarkably constant but the individuals who made up that population varied substantially. These data have to be considered when interpreting the studies described above that have classified individuals as having ACO based on a single bronchodilator test.

It would be helpful if patients with a positive BDR on one occasion behaved differently from those who did not but this does not seem to be true, at least in studies where patients did not have a history of prior asthma. The 4-year UPLIFT trial found no relationship between health status or exacerbation rate and the initial bronchodilator response [83]. This was confirmed in the ECLIPSE dataset [82]. The ECLIPSE investigators went on to look at the subset of patients who were consistently positive on testing over 3 years and compared them to those with consistently negative tests and found no difference in mortality, hospitalisation or exacerbation rates.

In summary, classification of individual patients as having an asthma–COPD overlap condition based on a single bronchodilator test is unreliable and influenced by the nature of the test conducted, the severity of pre-test lung function impairment, the way in which it is interpreted and between day fluctuations in ASM tone. How much of the apparent difference in behaviour at a group level is determined by a greater than anticipated improvement in FEV_1_ after a short-term bronchodilator test remains uncertain.

## 6. Therapeutic Implications of ACO

One of the main reasons to identify patients as having ACO would be to vary their treatment in order to reflect the presence of a presumed dual pathology and potential treatment approaches have been reviewed before [84]. At present there is no evidence base comparing treatment efficacy in individuals meeting any of the ACO definitions with those with ‘pure’ COPD. Indeed, it seems unlikely that important differences would emerge in patients selected on the basis of any of the composite definitions currently proposed. Among COPD patients it is clear that even those who do not exhibit a positive response still benefit from long-acting inhaled bronchodilator treatment in terms of improved exercise capacity and reduced degrees of exertional breathlessness [85]. Hence, it would be illogical to restrict the use of these treatments to those who met the ACO criteria.

The crucial drug class where a clear distinction might be helpful is in the use of anti-inflammatory drugs. The most studied class has been ICS and here prior belief seems to trump evidence. For many working in this field it has been an item of faith that inhaled corticosteroids are ineffective in COPD and hence they need an explanation for the large body of data that show that ICS, usually combined with a long-acting inhaled bronchodilator, can improve health status, decrease exacerbation frequency, decrease the rate of decline in FEV_1_ and prolong life in a large clinical trial population [86]. The suggestion that positive results reflect the presence of a ‘hidden’ asthmatic population overlapping with ‘pure’ COPD is not supported by re-analysis of the trial data [87]. However, one characteristic which is part of some definitions of ACO can properly be considered to be a treatable trait on which therapeutic choices about ICS use can be based.

As discussed above blood eosinophil counts have been proposed as a way to identify an ACO subtype of COPD. Airway eosinophilia has been studied in airways disease for almost 20 years mainly focusing on patients with asthma and reporting induced sputum data [88]. However, the relationship between induced sputum eosinophil counts and those in blood is weak in patients diagnosed with COPD [89]. The recognition that COPD patients in the highest tertile of the normal range of eosinophil counts experienced significantly fewer exacerbations when treated with ICS+LABA compared with LABA alone changed perceptions radically [90]. These data were confirmed in other data sets [91,92] as a better understanding emerged about how best to interpret the threshold where the beneficial effect of ICS on exacerbation frequency emerged. In general, this was dictated by the a priori likelihood of an exacerbation occurring and the amount of background bronchodilator treatment, with patients with a blood eosinophil count and a prior exacerbation history being likely to benefit from using ICS irrespective of the degree of concomitant therapy [93]. The extent of peripheral blood eosinophilia did not influence any positive effect of ICS on either FEV_1_ or health status, but there are retrospective data suggesting that patients with higher blood eosinophil counts have a reduction in lung function loss over time when treated with ICS [94]. Rather surprisingly, the same association between blood eosinophil count and the effect of treatment on exacerbations was seen with a different agent, roflumilast [95]. Like inhaled corticosteroids [96], this drug decreases the degree of eosinophilia seen in airway biopsies [97]. Further mechanistic studies explaining these effects are needed.

**Figure 3:**
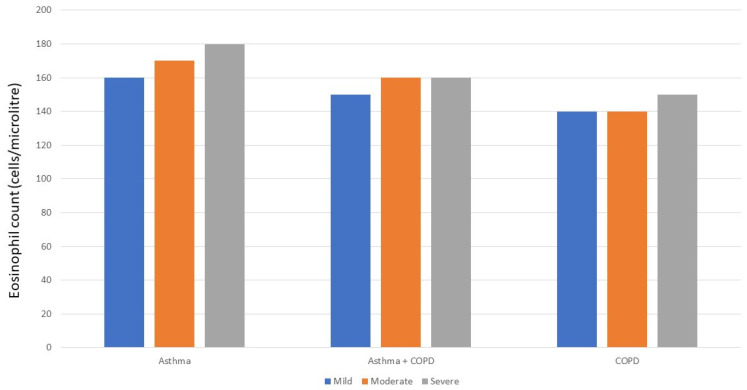
Mean absolute eosinophil count (cells/µL) in 11,243 patients who participated in the NOVELTY study which included 5940 with a physician diagnosis of asthma, 3907 with a physician diagnosis of COPD and 1396 with a physician diagnosis of asthma and COPD. The physician also assessed disease severity as mild, moderate or severe [51].

The problem for the ACO concept is that the presence of a higher blood eosinophil count is not related to other proposed features of an ACO diagnosis. The distribution of blood eosinophils in COPD populations is not different from that seen in healthy patients without the disease [98], suggesting that the coexistence of a higher count and COPD occurs by chance rather than due to a specific causal mechanism. As noted already, the blood eosinophil count in the large observational NOVELTY study was not different between patients with an ACO diagnosis and those thought to have usual COPD [51], a finding that held true across the clinician-determined range of disease severity (Figure 3). The diagnostic classification did have some significance as ACO patients were more likely to receive ICS treatment in disease perceived to be mild or moderately severe than was the case if COPD alone was diagnosed. However, a similar percentage of patients with severe disease received ICS and ICS+LAMA+LABA treatment irrespective of which diagnostic label was applied.

## 7. Conclusions

Doctors select medical labels for a variety of reasons—to explain to patients that their problems have a rational basis with predictable outcomes that are amenable to proven treatment, to indicate the likely clinical course and prognosis of the condition and finally, on some occasions, to conceal their diagnostic uncertainty and allow them freedom to select treatment that a more restrictive diagnosis would not necessarily allow. It is our view that most cases diagnosed as ACO fall into this last category. This is not due to any ill-intent on the part of the doctor but reflects the multidimensional way in which the diagnosis of asthma and COPD have been presented over the years with a lack of clarity about which features carry most weight in reaching a diagnostic conclusion and uncertainty about how the clinical manifestations of the illness relate to the pathological changes and disease mechanisms which cause them. It is now possible using more objective measurements made in life to categorise these processes differently but the ubiquity of both asthma and COPD, coupled with the long natural history of both conditions, make implementing this a challenging undertaking. Hence, we are likely to be left with composite diagnostic categories which will inform our clinical and academic approach to these conditions. This highlights the need for long-term cohort studies to better understand both the real-life trajectories of COPD and asthma over time, and to better identify phenotypes of patients who may experience features of COPD and asthma. The question remains in this setting—is ACO a useful diagnostic subdivision? As our review of the evidence suggests, we do not believe this is the case.

At the individual patient level, the lack of agreement about what a doctor might mean by the term ACO is a huge drawback. Extrapolating treatment algorithms for this condition based on what occurs in its better-defined progenitor conditions is not helpful. Reliance on a positive bronchodilator response means that the chance of the diagnosis being changed rises with the number of times the test is repeated, even when relatively strict definitions of a positive response are applied. Even if the response is positive, it does not preclude a useful response to the currently available inhaled therapies. Selection of patients based on relative blood eosinophilia has a better evidence base, at least for exacerbation prevention with some anti-inflammatory treatments. However, these beneficial effects are linked to the higher blood eosinophil count (which itself shows modest between day variation) rather than the other features of ACO and appear to be distributed across the general population rather than confined to a particular subset of patients with airways disease. In larger population studies, subjects identified as having ACO report more exacerbations than those with COPD alone. This may reflect the nature of their COPD pathology which increases their apparent AHR and leads to more between day fluctuation in airway calibre for reasons other than abnormal airway smooth muscle function.

Several approaches have been proposed to deal with these issues of classification. In a thoughtful article Wise and Putcha suggest four different ‘phenotypes’ of ACO (Table 4) which best explain associations between persistent airflow obstruction and asthma [99]. Like the other proposed definitions of ACO, there is a need for longitudinal data to prospectively determine the stability of the diagnostic groups and their subsequent outcomes. A more attractive approach is based on the approach of Agusti et al. who emphasise the treatable traits which may be present in an individual patient, where a high blood eosinophil count is seen as a biomarker of the ability of ICS to prevent exacerbations rather than a defining characteristic of a specific disease [100]. How widely this return to Orie’s chronic non-specific lung disease will be accepted remains to be seen. Like others who have reviewed this issue [17,18,19,33] we tend to the view that it is better to ascribe a dominant likely pathology and describe the individual features that need most attention (e.g., exertional dyspnoea, frequent exacerbations, weight issues, social impacts) rather than creating a separate disease category that follows a different treatment schedule of uncertain relevance to the patient’s needs.

Thus, the conclusion reached by the person who has thought most about this topic and advocated the renaissance of the term ACO in 2009, when they revisited this topic in 2015 [62], seems the most appropriate summary of the case against ACO independently of COPD:

“A precise and useful definition of asthma–COPD overlap has not been possible, and the condition itself appears to compromise several different sub-phenotypes. It is proposed that addressing disease components via a multidimensional approach to assessment and management of obstructive airway diseases will be useful to manage the heterogeneity of these conditions.”

## Figures and Tables

**Table 1:** A Consensus Definition of ACO proposed from an ERS Sponsored Round-table Discussion [19]. Diagnosis requires the presence of all 3 major criteria plus 1 minor criteria. LLN = lower limit of normal, BDR = bronchodilator reversibility.

Major Criteria	Minor Criteria
Persistent airflow limitation (post-bronchodilator FEV1/FVC <0.70 or LLN) in individuals 40 years of age or older; LLN is preferred At least 10 pack-years of tobacco smoking or equivalent indoor or outdoor air pollution exposure (e.g., biomass) Documented history of asthma before 40 years of age or BDR of >400 mL in FEV1	Documented history of atopy or allergic rhinitis BDR of FEV1 ≥200 mL and 12% from baseline values on 2 or more visits Peripheral blood eosinophil count of ≥300 cells/µL

**Table 2:** Selected studies reporting clinical features of people with ACO.

Study	Definition of ACO	Main Findings
Reddel et al. [52]	Physician diagnosis of asthma, COPD or both	12.4% asthma and COPD (ACO) More likely to smoke, higher blood neutrophil count, more breathless and poorer health status compared with asthma Earlier diagnosis, more upper airway disease compared with COPD Bronchodilator responsiveness and FeNO similar across groups
Morgan et al. [53]	Features of both: COPD—post-bd FEV1/FVC below LLN and Asthma—self report physician asthma diagnosis, use of asthma medication last year or wheezing last year	Prevalence of ACO 3.8% in LMIC residents People with ACO had more biomass fuel exposure, higher smoking and lower educational attainment Worse AFO than asthma or COPD groups
Toledo-Pons et al. [54]	Three groups: Diagnosed with asthma and COPD (smoking asthmatic) COPD and bronchial hyperresponsiveness (FEV1 increase >400 mL and 15%) (COPD high bronchial response) COPD and eosinophilia (eosinophils >300cells/µL) (COPD eosinophilia)	27.4% fulfilled one or more criteria for ACO 13.8% smoking asthmatic, 12.1% COPD with eosinophilia and 1.5% COPD with high bronchodilator response Smoking asthmatics were younger, more likely female and more atopic
Singh A et al. [55]	COPD—post-bronchodilator FEV1/FVC <0.7 Asthma—>200 mL and >12% improvement in FEV1 with bronchodilator ACO—both present	Prevalence of ACO 4.6% in firefighters Eosinophil count >300 cells/µL more common in ACO More likely to have accelerated decline in FEV1
Cosentino et al. [56]	ACO; either: history of asthma or hay fever, FEV1/FVC <0.7, >200 mL and >12% improvement in FEV1 with bronchodilator and less than 15% emphysema on CT, or FEV1/FVC <0.7, >400 mL and >15% improvement in FEV1 with bronchodilator and less than 15% emphysema on CT and less than 15% emphysema on CT regardless of history of asthma or hay fever	Compared to subjects with COPD and emphysema ACO subjects were younger, more likely African-American, higher BMI and more likely to still smoke
Krishnan et al. [57]	ACO defined as >40 years old, current or former smoker, FEV1/FVC <0.7 and >200 mL and >12% improvement in FEV1 with bronchodilator	Prevalence of ACO of 18.2% More common in people diagnosed with both asthma and COPD Younger and higher BMI compared with COPD cohort More likely to smoke and less rhinitis than asthma cohort
Izbicki et al. [58]	COPD was defined as FEV1 <80% predicted and FEV1/FVC <0.7. ACO was defined as this plus >200 mL and >12% improvement in FEV1 with bronchodilator	No differences seen compared with the COPD cohort except lower pre-bronchodilator lung function in ACO
Barrecheguren et al. [59]	ACO defined as COPD patients reporting a previous diagnosis of asthma Classified as ACO2 if had 2 major or 1 major & 2 minor criteria: Major criteria were improvement in FEV1 >400 mL and >15% with bronchodilator, sputum eosinophilia or a previous diagnosis of asthma before the age of 40 years Minor criteria were increased total serum immunoglobulin E, previous history of atopy or FEV1 >200 mL and >12% on two or more occasions	Prevalence of ACO of 15.9% Two thirds did not fulfil ACO2 criteria ACO subjects were more likely to be female, had more exacerbations, had better lung function and higher blood eosinophilia
Llanos et al. [60]	40 years old or greater with at least 1 asthma and 1 COPD characteristic: Asthma characteristic—even given a physician diagnosis of asthma or had an ‘asthma attack’ in the previous year COPD characteristic—post-bd FEV1/FVC <0.7 and ever told they had emphysema or chronic bronchitis by a physician	ACO subjects had poorer lung function than those with asthma or COPD, higher eosinophil counts than those with asthma or COPD and had more ‘asthma attacks’ than the asthma group
Baarnes et al. [61]	At least 1 previous hospitalisation for asthma and 1 for COPD	Subjects with ACO were older, more likely to smoke, had lower educational attainment and took less regular exercise

**Table 3:** Problems when interpreting bronchodilator responsiveness in people with COPD [73].

Pitfall with Reversibility Testing	Reason for the Problem
The bronchodilator drug used	Additional bronchodilation with the combination of short-acting beta-agonists and short-acting anti-muscarinics compared to one bronchodilator
The timing of reversibility testing	Short-acting anti-muscarinics achieve maximum bronchodilation longer than 15 min after administration, the timing typically used for beta-agonist reversibility
The dose of bronchodilator drug	Higher doses of salbutamol (>400 mcg) will result in further small increases in FEV1 compared with lower doses
The reproducibility of result	The magnitude of reversibility, and classification of reversibility (positive or negative), varies significantly between tests
The impact of pre-test FEV1	Individuals with a lower pre-test FEV1 are less likely to shown significant reversibility
The clinical implications of reversibility	Reversibility does not predict clinical symptoms, exacerbations and subsequent decline in lung function

**Table 4:** A suggestion for different pathways to ACO presented as 4 different ‘phenotypes’ of ACO described by Putcha and Wise [99].

Phenotype of ACO	Clinical and Biological Features
Smokers with airflow obstruction and eosinophilic inflammation	Exacerbations driven by eosinophilic inflammation Better lung function, less emphysema, less disease progression Better response to oral and inhaled corticosteroids Higher level of atopy
Resistant asthmatic	Asthmatics less responsive to corticosteroids Higher level of irreversible airflow obstruction Neutrophil dominated airway inflammation and exacerbations are more common
Elderly asthmatic with irreversible airflow obstruction	Long-standing asthma and irreversible airflow obstruction Neutrophil dominated airway inflammation Loss of lung elastic recoil and more hyperinflation
Childhood asthmatic who smokes and has developed COPD	Asthma as child or young adult but long-term smoking Higher number of pack years (more likely to have >20 pack years) High symptom burden and healthcare utilisation

## Data Availability

Not applicable.
